# Effects of empowerment education on the self-management and self-efficacy of liver transplant patients: a randomized controlled trial

**DOI:** 10.1186/s12912-023-01298-6

**Published:** 2023-04-28

**Authors:** Limin Guo, Lezhi Li, Yanfang Lu, Ting Li, Linjun Chen, Liya Jiang, Shihan Zhang, Meijiao Yuan

**Affiliations:** 1 Clinical Nursing Teaching and Research Section, The Second Xiangya Hospital, Central South University, Changsha, 410011 China; 2 Liver transplant department, The Second Xiangya Hospital, Central South University, Changsha, China

**Keywords:** Liver transplant patients, Empowerment education, Self-management, Self-efficacy, RCT

## Abstract

**Background:**

Despite the increasing survival rates, liver transplant patients experience numerous postoperative complications and encounter significant challenges in long-term self-management. This study aims to examine the effectiveness of empowerment education in enhancing self-management skills and self-efficacy among liver transplant recipients.

**Methods:**

A randomized, single-blind, single-center trial was conducted in China between August 2019 and September 2020, involving liver transplant recipients. The intervention group received 12 weeks of empowerment education, while the control group received 12 weeks of routine education. .The study assessed the patients’ self-management and self-efficacy using the Liver Transplant Recipient Self-Management Questionnaire and the Self-efficacy for Managing Chronic Disease 6-Item Scale. Follow-up assessments were conducted at 1, 3, and 6 months after the intervention.

**Results:**

Eighty-four patients were initially randomized to either the intervention group (n1 = 42) or the routine education group (n2 = 42). Twelve patients were excluded from the analysis due to loss of follow-up or discontinuation of the intervention, leaving 72 patients (n1 = 35, n2 = 37) for the final analysis. The scores for exercise and lifestyle management were significantly higher in the intervention group than in the control group at 1, 3, and 6 months after the intervention (t = 3.047, 5.875, 8.356, and t = 5.759, 4.681, 11.759, respectively; *P* < 0.05). At 3 and 6 months after the intervention, the scores for cognitive symptom management, communication with physicians, and self-efficacy were significantly higher in the intervention group than in the control group (t = 5.609, 6.416, and t = 5.576, 11.601, and t = 6.867, 15.071, respectively; *P* < 0.001). Within the intervention group, self-management scores increased significantly over time, while within the control group, the scores for communication with physicians, lifestyle, and self-efficacy showed a significant decline from 3 to 6 months after routine health education.

**Conclusions:**

The results of this study suggest that empowerment education is an effective means of improving the self-management and self-efficacy of liver transplant patients, with better outcomes compared to routine health education. These findings have important implications for nursing practice and provide valuable guidance for clinical education of liver transplant patients.

**Trial registration:**

ChiCTR2200061561.

## Background

Liver cirrhosis is a pathological condition that is characterized by chronic liver inflammation, diffuse fibrosis, pseudolobules, regenerative nodules, and vascular proliferation within and outside of the liver. During the compensated stage, patients may not exhibit obvious symptoms, but in the decompensated stage, patients often experience portal hypertension and a decrease in liver function. Liver transplantation is currently the only effective treatment option for end-stage liver disease. This procedure has been performed for over 50 years, and as of 2017, more than 100,000 liver transplantations have been conducted worldwide [1]. As of June 2019, the China Liver Transplant Registry (CLTR) reported that nearly 40,000 liver transplantations have been performed in China. The 5-year and 10-year survival rates for liver transplant patients in China are reported to be 80% and 70%, respectively. [2].

Self-management refers to the individual’s ability to manage the symptoms, treatment, physical and psychosocial consequences and lifestyle changes inherent in living with a chronic condition [3]. Despite the high survival rates, liver transplant patients in China exhibit low levels of self-management [4, 5]. Previous research has demonstrated that practicing self-management can lead to an improvement in both physical and mental health, as well as an enhanced quality of life and decreased healthcare costs [6, 7]. However, self-management can be influenced by several factors, with self-efficacy being one of the most significant [8]. Self-efficacy is an individual’s expectation of whether they have the ability to perform a certain behaviour and people’s cognition and evaluation of self-behaviour ability [9]. Self-efficacy is known to be closely related to patient treatment compliance, self-management behaviours, and social support [10, 11]. Therefore, it is important to explore an effective way to improve the self-management and self-efficacy of liver transplant patients.

Several studies have indicated that liver transplant recipients have a lower-middle level of self-management.[4, 5]. Liver transplant recipients have to take immunosuppressive drugs for the rest of their lives after the surgery and may suffer from various postoperative complications, such as graft failure, infections, neoplasms, metabolic syndrome, and surgical complications, which make them chronically ill for a prolonged period [12]. Studies have revealed that a significant number of liver transplant patients, ranging from 20 to 40%, experience rejection after surgery, which is linked to timely review of drug concentrations [13]. Moreover, approximately 10–45% of liver transplant patients revert to unhealthy habits, such as alcohol consumption, smoking, and irregular sleep patterns, leading to reduced survival rates [14, 15]. It is evident that the self-management level of liver transplant patients is closely associated with their quality of life. In this regard, self-efficacy plays a crucial role in shaping self-management behaviours [16].

It is important to note that while different educational methods have been used to improve the self-management ability of liver transplant patients, such as self-management education, continuous nursing, and health management, they are often formulated by medical staff based on the patient’s condition and tend to be guided and didactic in nature [17–19]. The long-term effects of these methods on patient compliance and self-management ability are not always evident.

Funnell’s proposition that empowerment can help patients identify internal problems and improve their self-management ability through relevant measures [20] has led to the development of empowerment education, which is based on the theory of self-determination (SDT) and autonomy support [21]. This patient-centered approach respects the patient’s decision-making and related actions, enhances their autonomy, and allows them to assume corresponding responsibilities while receiving routine education. In traditional medical education, the medical staff takes the lead and patients receive information and instructions passively. However, the authorization theory emphasizes that patients should take full responsibility for their own self-management [20]. Patients’ living habits are mainly changed by the individual themselves, with the assistance of medical staff. In addition, they are fully responsible for their lives after actively listening to the suggestions of the medical staff [22]. In 2008, Mou Lining introduced and applied the authorization theory to diabetic patients in China, which resulted in statistically significant differences in blood glucose, BMI, and blood pressure [23]. Since then, researchers have used empowerment education to improve the self-management, self-efficacy, and psychological coherence of patients with chronic diseases, and significant results have been reported in several studies [24–26].

In summary, the self-management of liver transplant recipients is at a low-middle level. Different educational methods, such as self-management education, continuous nursing and health management, are educator-centred, which have limited effects on liver transplant patient compliance and self-management. Since empowerment education is patient-centred, it can enhance patients’ autonomy and responsibility, and its long-term effects have been proven. Therefore, this study aims to verify that empowerment education has a better effect on self-management and self-efficacy in liver transplant patients than routine education. Two hypotheses are proposed: (1) empowerment education can effectively improve the self-management and self-efficacy of liver transplant patients; (2) the effect of empowerment education is better than routine education.

## Methods

### Aim, design and setting of the study

The aim of this intervention is to enhance the self-management and self-efficacy of liver transplant patients through a randomized, single-blind, single-center trial. The study enrolled liver transplant patients who were admitted to the Second Xiangya Hospital between August 2019 and September 2020, and randomization was performed using a central computer system. A total of 84 eligible liver transplant patients were assigned to two groups in a 1:1 allocation ratio. The Second Xiangya Hospital is a general hospital that serves individuals from all socioeconomic backgrounds in the local community.

### Characteristics of participants

The study included liver transplant patients who met the following inclusion criteria: (a) having indications for liver transplantation and have received orthotopic liver transplantation; (b) over 18 years of age, with stable postoperative conditions and no serious complications that did not affect their daily activities; (c) ability of oral and verbal communication; and (d) provided informed consent for voluntary participation in this study. The exclusion criteria were: (a) previous liver or other organ transplantation; (b) severe heart, lung and kidney diseases; and (c) somatic activity disorder that would significantly impact the exercise measures. Participants who experienced disease aggravation, died, dropped out, or were lost to follow-up during the intervention were excluded from the analysis.

The researchers enrolled 84 participants who were numbered in advance. Then, a random number generator was used to generate corresponding random numbers in a 1:1 ratio. Participants with odd random numbers were assigned to group A and even random numbers were assigned to group B by the corresponding author, who was blind to the specific grouping situation (group A as intervention group and group B as control group). Data analysts and collectors were also blinded to the group allocations.

### Theoretical framework

**Fig. 1:**
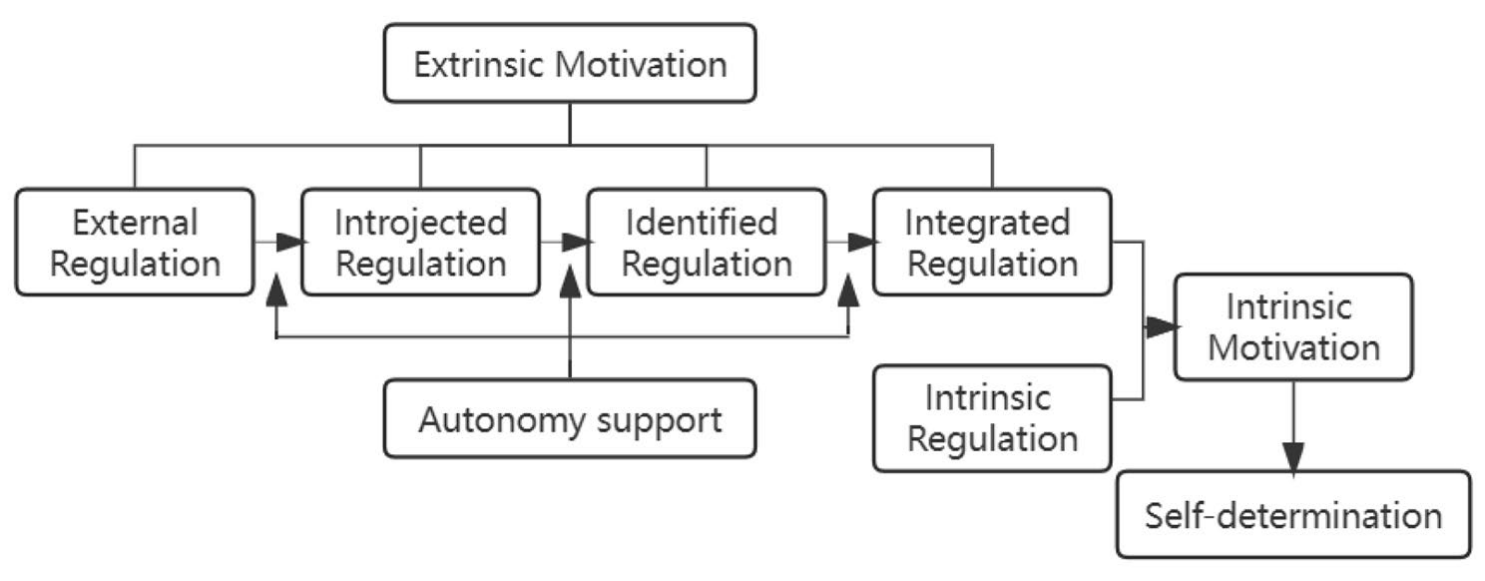
Theoretical framework

Empowerment education was developed based on the theory of self-determination (SDT) and autonomy support [21]. Self-determination theory distinguishes motivation as either intrinsic or extrinsic. Extrinsic motivation encompasses external regulation, introjected regulation, identified regulation, and integrated regulation. These four types of regulation are positioned on a continuum with different levels of internalization. Empowerment education aims to support patient autonomy through a five-step process: problem identification, emotional expression, goal setting, plan development, and outcome evaluation. When patients’ needs for autonomy, competence, and relatedness are met, this promotes a shift from external regulation to internalization, where integrated and intrinsic regulations work together to form intrinsic motivation towards adopting a healthy lifestyle and improving quality of life. The theoretical framework is presented in Fig. 1.

### Intervention

#### Establishment of the empowerment education group

The group consisted of eight medical staff members, including one associate professor, one head nurse, three supervisor nurses, and three primary nurses. The associate professor has over 10 years of experience in liver transplantation surgery, a doctoral degree, and research experience. The head nurse has more than 15 years of experience in liver transplant nursing, a postgraduate degree, and research experience. The supervisor nurses and primary nurses have more than 5 years of experience in liver transplant nursing and a bachelor’s degree.

Two intervention nurses received training in delivering empowerment education during four two-hour face-to-face sessions conducted by the researcher. Prior to the intervention, the intervention nurse answers open-ended questions regarding the content and methodology of empowerment education. The intervention was delivered systematically according to the protocol, and after each session, the intervention nurse recorded the length and content of the intervention. To ensure proper execution of the study, the researchers held weekly meetings to review progress.

#### Implementation of the intervention

The intervention group received 12-week (2 face-to-face group sessions weekly for the first month and weekly telephone calls for the last two months) empowerment education based on the liver transplantation handbook. The liver transplantation handbook was revised by a liver transplantation specialist, and it includes basic information about liver transplantation, postoperative dietary guidance, medication, pipeline, skin, exercise, complications, psychological adjustment, and follow-up. Empowerment education contains five steps: (1) establish problems–open-ended questions are used to guide patients to find the main problems; (2) express emotions–encourage patients to vent their feelings and listen carefully; (3) set goals–guide patients to make their own suitable goals according to their problems; (4) develop plans–the intervention implementer provides relevant knowledge of the existing problems and guides patients to propose plans for solving problems; and (5) evaluate effects–ask the patient to elaborate on how the plan is being implemented and what problems exist. The effect evaluation was conducted before the next group session, and the order of the five steps of empowerment education was not fixed. The curriculum covered all five steps, and if new problems were proposed by the patients during the implementation process, these could be given priority even if previous problems were already in the planning or other stage.

### Control group

The control group underwent 12 weeks of routine education and follow-up conducted by three nurses. The routine education consisted of two face-to-face group sessions per week, each lasting 30 min, on the same topics covered in the empowerment education program. This was followed by weekly telephone calls over two months to provide additional health counseling. No empowerment strategies were included in the routine education.

### Quality control

Study quality control measures included:

- Strictly following the inclusion and exclusion criteria to minimize selection bias and ensure comparability of the data for research objects.
- Providing uniform professional training to all investigators and intervention implementers.
- Arranging the intervention group patients in wards far away from the control group to reduce contamination.
- Establishing a database using Epidata 3.1 with double entry, where all data was entered in pairs and checked for consistency. Any inconsistent paired data was verified until 100% consistency was achieved.

### Measurements

#### Liver transplant recipient self-management questionnaire

The questionnaire used in the study was adapted from the Chinese version of the “Chronic Disease Self-Management Program Questionnaire Code Book,“ originally developed by Dr. Lorig et al. from the Stanford Patient Education Research Center, by Xing et al. [27]. The self-management assessment consisted of four subcategories, including exercise, cognitive symptom management, communication with physicians, and lifestyle management. Exercise was measured by the duration (in minutes per week) of exercise performed. Participants rated their exercise level using a five-point Likert scale: 0 for no exercise, 1 for < 30 min per week, 2 for 30–59 min per week, 3 for 1–3 h per week, and 4 for > 3 h per week. Cognitive symptom management was evaluated using a five-item scale, with each item rated on a six-point scale ranging from 0 to 5, anchored by “none of the time” and “all of the time”. This scale aimed to assess patients’ ability to deal with changes under different conditions. The final score for cognitive symptom management was calculated as the mean of the five items, with higher scores indicating a greater use of cognitive techniques. The communication ability of LT patients with physicians was assessed using a four-item scale, with each item rated on a six-point scale (0–5) anchored by “never” and “always”. The score for communication with physicians was calculated as the mean of the four items, with higher scores indicating better communication with physicians. Lifestyle management was assessed using a thirty-item scale, with each item rated on an eight-point scale (1–8) anchored by “no influence” and “influence as great as it could be”. The score for lifestyle management was the mean of the thirty items, with higher scores indicating greater achievement in establishing a healthy lifestyle that meets the needs of the disease and therapy. The questionnaire had a Cronbach’s coefficient of 0.87 [27] and was used to assess the level of self-management among liver transplant patients. A scoring indicator of ≤ 60% was considered “less than satisfactory”, while a score of > 60% was considered “good” based on the total score indicator and the average of the four subcategory scoring indicators.

#### Self-efficacy for managing chronic disease 6-item scale

The scale was originally developed by Lorig et al. and consisted of six items, with each item scored on a scale of 1 (not at all confident) to 10 (totally confident) [28]. The total score of the scale is the average of the six items, with higher scores indicating higher levels of self-efficacy. Based on the scoring indicators, self-efficacy levels were categorized as high, medium, and low. A score of ≥ 80% indicated a high level of self-efficacy, 60–80% indicated medium level, and ≤ 60% indicated a low level. While there is currently no specific self-efficacy scale for liver transplant patients, this scale was found to have a Cronbach’s coefficient of 0.87 in liver transplant patients [27] and was therefore used in this study to assess self-efficacy levels.

### Data collection

The demographic, self-management, and self-efficacy data were collected from liver transplant patients once they were transferred from the ICU. The self-management and self-efficacy data were collected at 1, 3, and 6 months after the intervention. The data collection was carried out by nurses who were not part of the research team.

### Statistical analysis

After data entry, statistical analysis was performed using SPSS 25.0 [29]. Descriptive statistics were used to present measurement data as mean ± standard deviation, and count data as frequency and percentage. The chi-square test and t-test were used to compare between-group differences. Normality was tested using frequency distribution histograms, and all data were found to conform to a normal distribution. Two-factor repeated-measures analysis of variance (ANOVA) was used to compare the self-management and self-efficacy scores between the intervention and control groups over time. Pairwise comparisons were conducted using the Bonferroni method at different time points within each group, with a significance level of P < 0.05. The analysis was conducted according to the study protocol.

### Sample size

Based on previous findings on empowerment education [30], the standard deviation σ = 15.90, and the mean difference δ = 12.55. The sample size calculation formula used was as follows:

$${n_1} = {n_2} = 2\left[ {\frac{{{u_\alpha } + {u_\beta }}}{{\delta /\sigma }}} \right]^2 + \frac{1}{4}{u_\alpha }^2$$

Assuming a significance level of α = 0.05 and a power of 1-β = 0.8, u_α/2_=1.96, and u_β_=1.282. Thus, a sample size of 84 cases was required with n1 = n2 = 35 after considering a 20% loss ratio of follow-up.

## Results

### Baseline characteristics

**Fig. 2:**
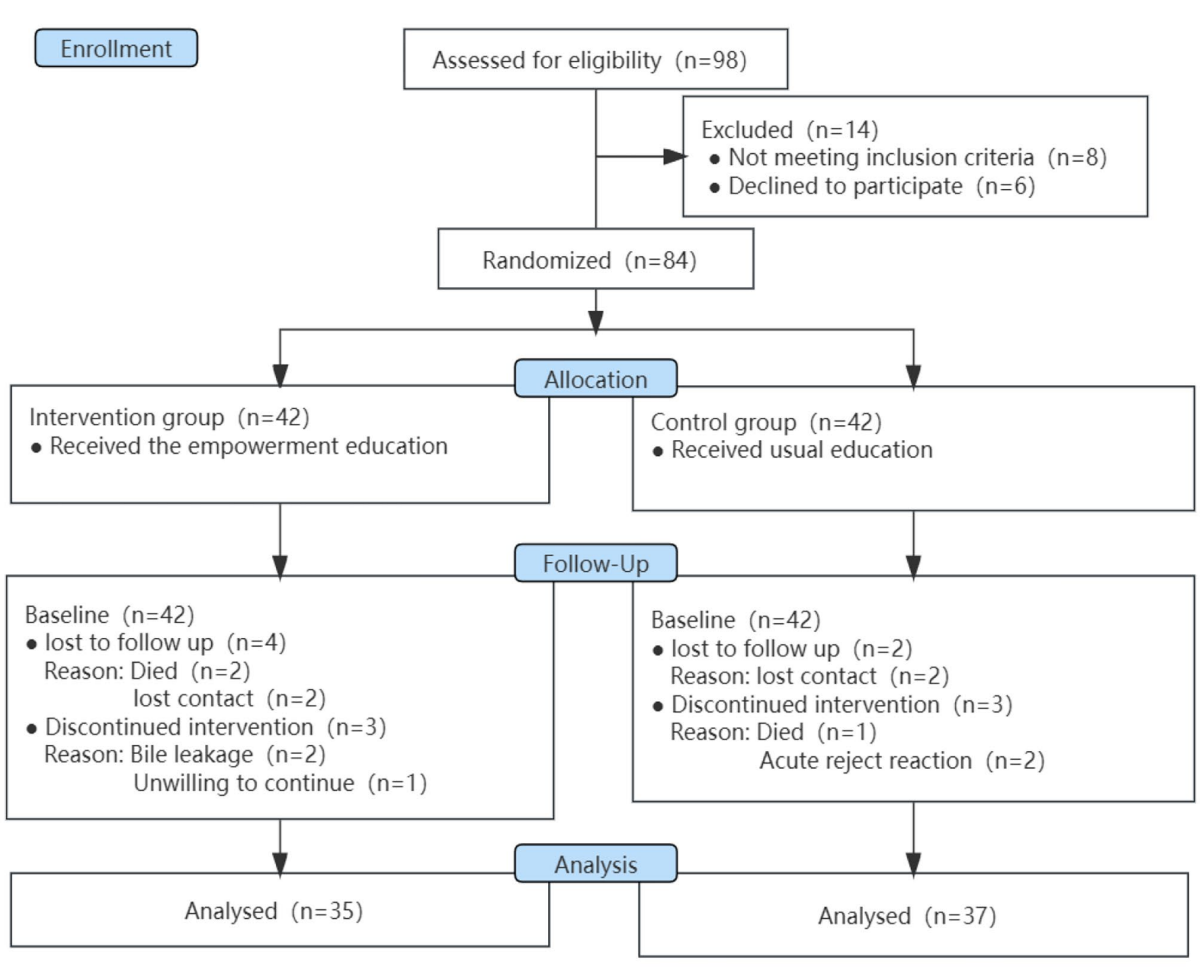
Flowchart of the data collection procedure

In this study, initially, 42 patients per group were included. However, during the study, 12 cases were lost (7 cases in the intervention group and 5 cases in the control group), resulting in a loss rate of 14.29%, shown in Fig. 2. Ultimately, 35 patients in the intervention group and 37 patients in the control group completed the study. There were no significant differences in demographic characteristics between the two groups (*P* > 0.05), indicating that the groups were comparable, as shown in Table 1. Additionally, mobility and other side effects were comparable between the two groups.

**Table 1:** Baseline demographics of the study sample by group

Variables	EEG(n = 35) n (%)	CG(n = 37) n (%)	*t*	χ^2^	*P*
Age (M ± SD)	47.63 ± 11.78	48.70 ± 10.75	− 0.404		0.687^a^
Gender				2.794	0.095^b^
Male	31(88.6)	27(73.0)			
Female	4(11.4)	10(27.0)			
Residence				2.022	0.155^b^
City	21(60.0)	16(43.2)			
Rural areas	14(40.0)	21(56.8)			
Marital status				0.278	0.598^b^
Married	30(85.7)	30(81.1)			
Widowed	5(14.3)	7(18.9)			
Years of education				0.196	0.658^b^
≤ 9	19(54.3)	22(59.5)			
≤9	16(45.7)	15(40.5)			
Income (CNY)				0.695	0.706^b^
≤ 3000	15(42.9)	17(45.9)			
3001 ~ 6000	15(42.9)	17(45.9)			
≤6000	5(14.2)	3( 8.2)			
Occupation status				1.479	0.477^b^
Currently working	17(48.6)	13(35.1)			
Retired	3( 8.6)	3( 8.1)			
Unemployed	15(42.8)	21(56.8)			
Disease duration since onset (years)				4.351	0.114^b^
≤ 1	8(22.9)	15(40.5)			
1–3	8(22.9)	3( 8.1)			
≤3	19(54.2)	19(51.4)			

Abbreviations: EEG, empowerment education group; CG, control group; χ2,chi-square test; M ± SD: mean ± standard deviation; CNY, Chinese Yuan

a Independent t-test

b Fisher’s exact-test

### Main outcomes

The results of the two-factor repeated-measures analysis of variance indicated that there were statistically significant differences between the two groups in terms of exercise, cognitive symptom management, communication with physicians, lifestyle management, and self-efficacy across the three time points (*P* < 0.001), as shown in Table 2. Furthermore, there was a significant interaction effect between time and group, indicating that the effect of time on the outcomes varied depending on the group. To further investigate this interaction effect, pairwise comparisons using the Bonferroni method were conducted.

Pairwise comparisons between groups at different time points showed that there were no statistically significant differences in exercise, cognitive symptom management, communication with physicians, lifestyle management and self-efficacy between the two groups before the intervention (t = -0.274, t = -0.033, t = 0.097, t = 1.347, t = -0.728, *P* > 0.05). However, at 1, 3 and 6 months after the intervention, the intervention group showed significantly higher scores in exercise and lifestyle management compared to the control group (t = 3.047 vs. 5.875 vs. 8.356, t = 5.759 vs. 4.681vs. 11.759, *P* < 0.05). Additionally, the scores of cognitive symptom management, communication with physicians, and self-efficacy in the intervention group were significantly higher than those in the control group at 3 and 6 months after the intervention (t = 5.609 vs. 6.416, t = 5.576 vs. 11.601, t = 6.867 vs. 15.071, *P* < 0.001) (Table 3).

**Fig. 3:**
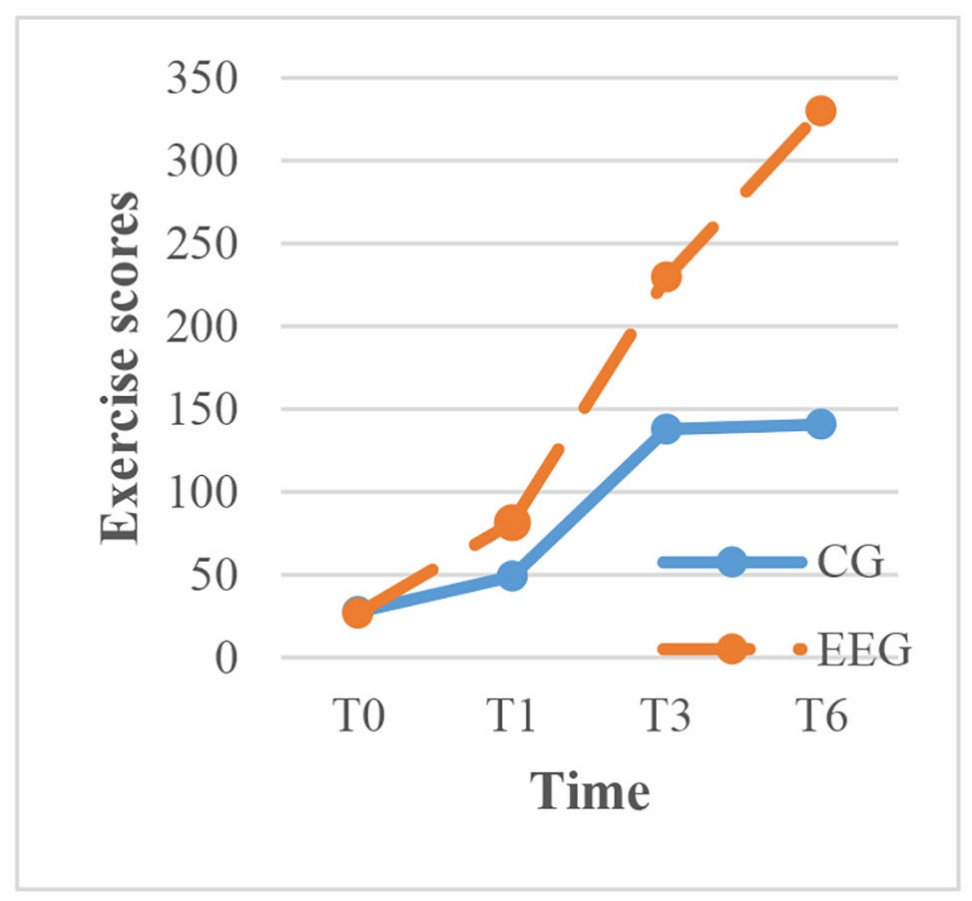
The trend of exercise scores before and after intervention in the two groups. CG, control group; EEG, empowerment education group. TIME: T0, before the CG, control group; EEG, empowerment education group. TIME: T0, before the intervention; T1, one month after intervention; T3, three months after intervention; intervention; T1, one month after intervention; T3, three months after intervention; T6, six months after intervention. T6, six months after intervention

**Fig. 4:**
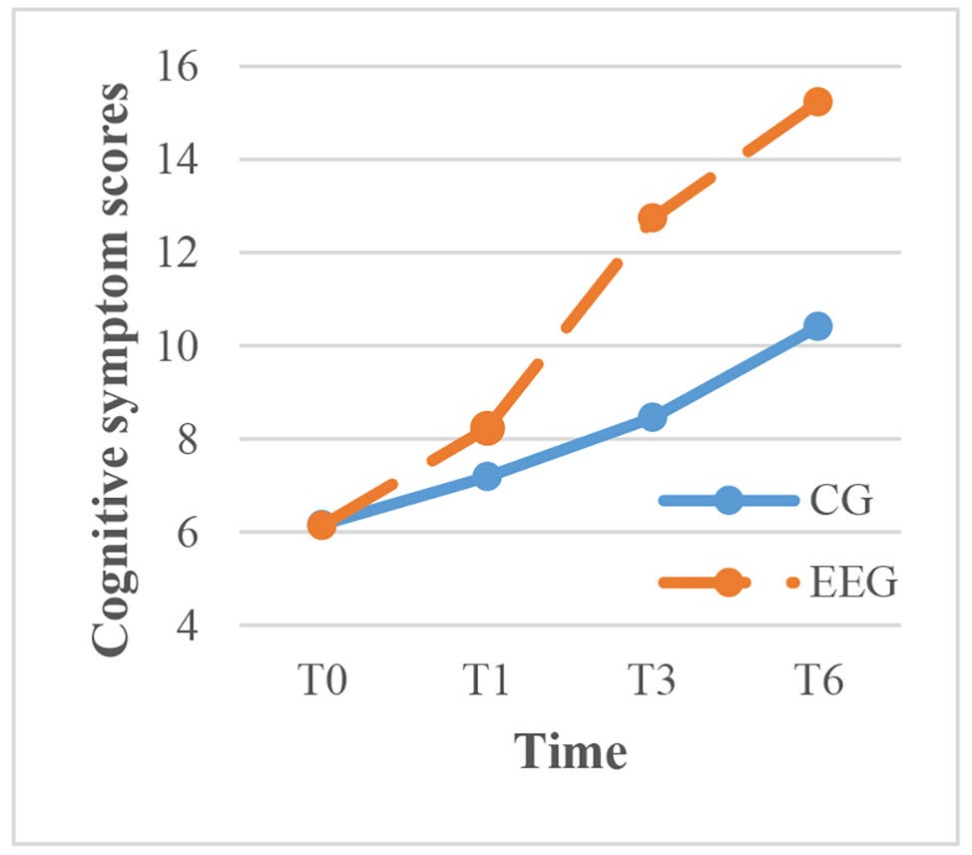
The trend of cognitive symptom scores before and after intervention in the two groups. TIME: T0, before the CG, control group; EEG, empowerment education group. TIME: T0, before the intervention; T1, one month after intervention; T3, three months after intervention; intervention; T1, one month after intervention; T3, three months after intervention; T6, six months after intervention. T6, six months after intervention

**Fig. 5:**
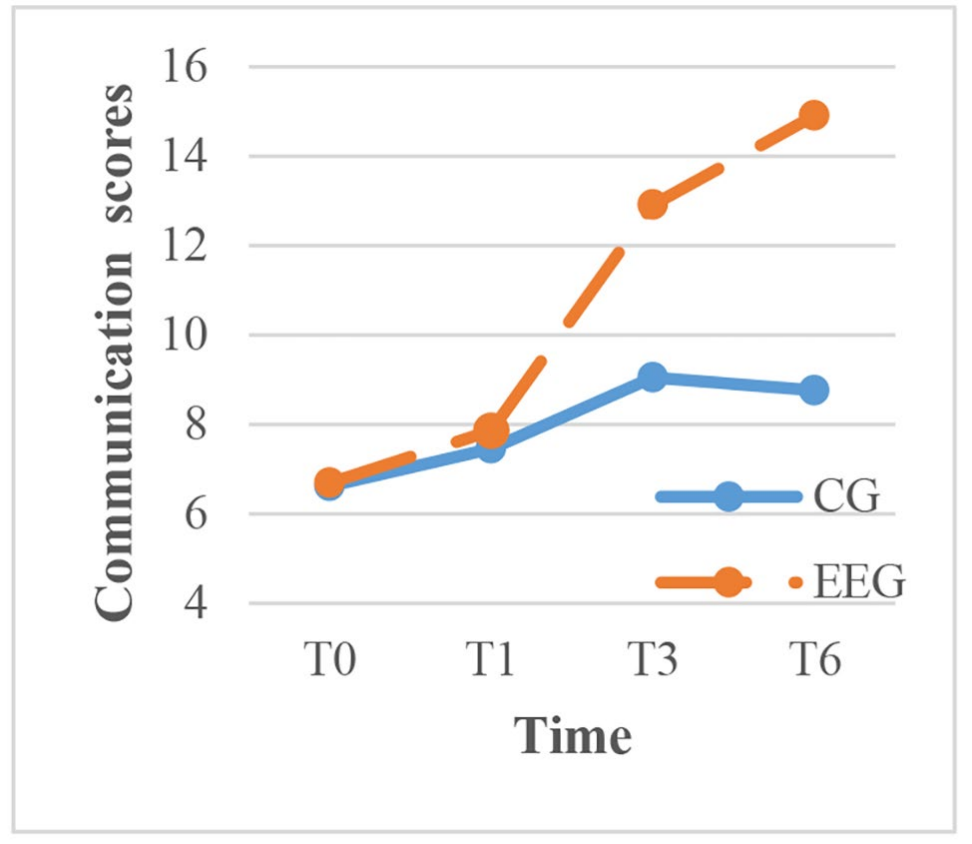
The trend of communication scores before and after intervention in the two groups. CG, control group; EEG, empowerment education group. TIME: T0, before the intervention; T1, one month after intervention; T3, three months after intervention; T6, six months after intervention

**Fig. 6:**
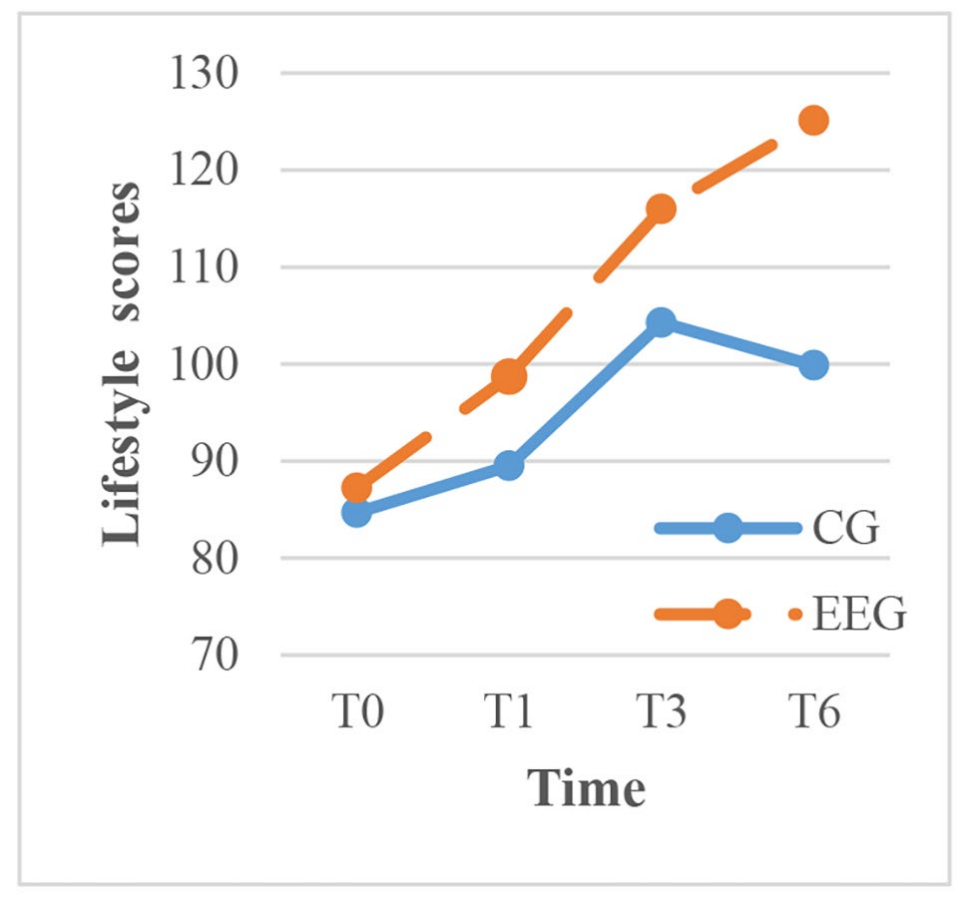
The trend of lifestyle scores before and after intervention in the two groups. CG, control group; EEG, empowerment education group. TIME: T0, before the intervention; T1, one month after intervention; T3, three months after intervention; T6, six months after intervention

**Fig. 7:**
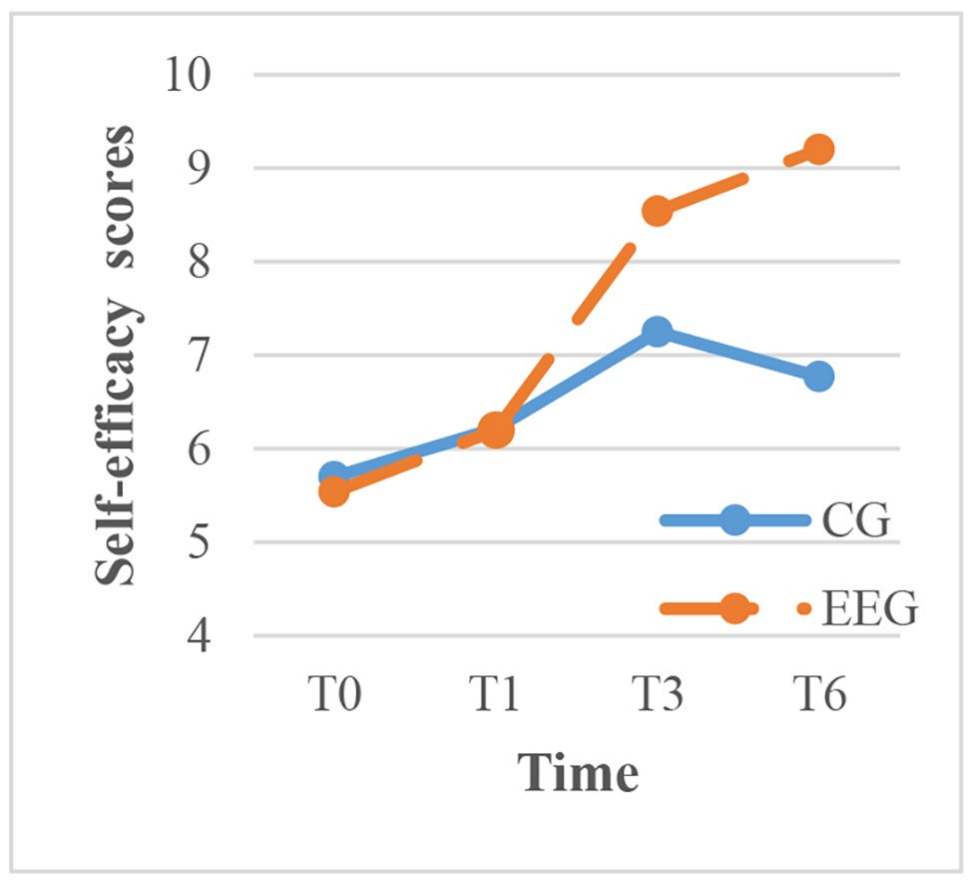
The trend of self-efficacy scores before and after intervention in the two groups. CG, control group; EEG, empowerment education group. TIME: T0, before the intervention; T1, one month after intervention; T3, three months after intervention; T6, six months after intervention

To clarify the results, within-group comparisons were made for each time point. The intervention group showed significant improvement in exercise, cognitive symptom management, communication with physicians, and lifestyle management over time (P < 0.001), with the most rapid improvement occurring between 1 and 3 months after the intervention. In contrast, the control group showed significant improvement in cognitive symptom management over time, but the scores for communication with physicians, lifestyle management, and self-efficacy decreased between 3 and 6 months after routine health education (Figs. 3–7).

During the trial, 4 patients in the empowerment education group and 9 patients in the control group developed complications. There was no significant difference in the complication rate between the two groups (χ2 = 0.033, *P* = 0.855).

## Discussion

To the best of our knowledge, this study is the first randomized controlled trial to examine the effectiveness of EE in self-management and self-efficacy among liver transplant patients. The findings of our study support the validity of our two hypotheses, which propose that EE is a highly effective intervention for enhancing self-management and self-efficacy among liver transplant patients, and that its effectiveness surpasses that of routine health education. Overall, these results highlight the clinical significance and robustness of this intervention.

**Table 2:** Two-factor repeated measures analysis of self-management and self-efficacy between the two groups before and after intervention

Variables		Before intervention	One month after intervention	Three months after intervention	Six months after intervention	F1(*P*)	F2(*P*)	F3(*P*)
Exercise	EEG	26.57 ± 17.85	81.43 ± 49.25	229.71 ± 83.82	330.00 ± 129.04	192.909^***^	76.691^***^	29.030^***^
	CG	27.97 ± 24.79	49.05 ± 40.72	137.84 ± 40.29	140.68 ± 37.31			
Cognitive symptom management	EEG	6.14 ± 2.76	8.23 ± 2.07	12.74 ± 4.00	15.23 ± 4.02	92.170^***^	27.458^***^	16.469^***^
	CG	6.16 ± 2.23	7.19 ± 2.38	8.46 ± 2.16	10.41 ± 1.95			
Communication with physicians	EEG	6.69 ± 2.82	7.86 ± 2.68	12.91 ± 3.43	14.91 ± 2.71	125.740^***^	30.687^***^	42.312^***^
	CG	6.62 ± 2.80	7.46 ± 2.06	9.05 ± 2.30	8.76 ± 1.64			
Lifestyle management	EEG	87.23 ± 8.68	98.71 ± 5.70	115.97 ± 11.83	125.11 ± 10.69	109.227^***^	95.845^***^	22.892^***^
	CG	84.68 ± 7.39	89.49 ± 7.79	104.27 ± 9.30	99.86 ± 7.05			
Self-efficacy	EEG	5.54 ± 0.90	5.54 ± 0.90	5.54 ± 0.90	5.54 ± 0.90	141.138^***^	36.869^***^	35.197^***^
	CG	5.70 ± 1.00	5.70 ± 1.00	5.70 ± 1.00	5.70 ± 1.00			

Abbreviations: EEG, empowerment education group; CG, control group; F1, time; F2, group; F3, time and group; ^***^P < 0.001

**Table 3:** Pairwise comparisons of self-management and self-efficacy between the two groups before and after intervention

Variables		Before intervention	One month after intervention	Three months after intervention	Six months after intervention
Exercise	EEG	26.57 ± 17.85	81.43 ± 49.25^a^	229.71 ± 83.82^ab^	330.00 ± 129.04^abc^
	CG	27.97 ± 24.79	49.05 ± 40.72^a^	137.84 ± 40.29^ab^	140.68 ± 37.31^ab^
t		-0.274	3.047	5.875	8.356
p		0.785	0.003	<0.001	<0.001
Cognitive symptom management	EEG	6.14 ± 2.76	8.23 ± 2.07^a^	12.74 ± 4.00^ab^	15.23 ± 4.02^abc^
	CG	6.16 ± 2.23	7.19 ± 2.38^a^	8.46 ± 2.16^ab^	10.41 ± 1.95^abc^
t		-0.033	1.972	5.609	6.416
p		0.974	0.053	<0.001	<0.001
Communication with physicians	EEG	6.69 ± 2.82	7.86 ± 2.68^a^	12.91 ± 3.43^ab^	14.91 ± 2.71^abc^
	CG	6.62 ± 2.80	7.46 ± 2.06^a^	9.05 ± 2.30^ab^	8.76 ± 1.64^ab^
t		0.097	0.708	5.576	11.601
p		0.923	0.481	<0.001	<0.001
Lifestyle management	EEG	87.23 ± 8.68	98.71 ± 5.70^a^	115.97 ± 11.83^ab^	125.11 ± 10.69^abc^
	CG	84.68 ± 7.39	89.49 ± 7.79^a^	104.27 ± 9.30^ab^	99.86 ± 7.05^abc^
t		1.347	5.759	4.681	11.759
p		0.182	<0.001	<0.001	<0.001
Self-efficacy	EEG	5.54 ± 0.90	6.20 ± 0.87^a^	8.54 ± 0.66^ab^	9.20 ± 0.60^abc^
	CG	5.70 ± 1.00	6.22 ± 0.96^a^	7.25 ± 0.91^ab^	6.77 ± 0.75^abc^
t		-0.728	0.498	6.867	15.071
p		0.469	0.941	<0.001	<0.001

Abbreviations: EEG, empowerment education group; CG, control group; F1, time; F2, group; F3, time and group;^***^^a^ comparison with pre-intervention,*P* < 0.05; ^ab^ compared with the 1 month after the intervention, *P* < 0.05; ^abc^ compared with the 3 month after the intervention, *P* < 0.05

The results of the present study demonstrated that the self-management gradually increased over time in the intervention group. These findings are consistent with previous studies indicating that EE can enhance patients’ disease awareness and self-care ability [30, 31]. Furthermore, our data revealed that the effect of EE is superior to that of routine health education. Although routine health education can improve the self-management of liver transplant patients, its effect is short-lived. Conversely, through the process of receiving EE, patients gradually internalize external motivation, develop self-decision-making ability, enhance their sense of disease control, and ultimately improve their disease management [21]. In addition, EE emphasizes autonomy support [32], which means ‘an individual in a position of authority takes the other’s perspective, acknowledges the other’s feelings, and provides the other with pertinent information and opportunities for choice, while minimizing the use of pressures and demands’[33]. When healthcare providers adopt an autonomy-supportive style, patients tend to exhibit greater intrinsic motivation to follow their medication regimen and have greater belief in the rationality of their prescriptions. The benefits of EE have also been observed in patients with heart failure, diabetes, and stroke undergoing rehabilitation [34–37].

Moreover, our study found that liver transplant patients had lower self-efficacy scores (5.62 ± 0.96) compared to other patient populations [10, 37]. This suggests that liver transplant patients may experience more difficulty in achieving positive psychological changes compared to other patients, as they may be coping with surgery scars and drainage tubes, which can affect their confidence in managing their disease and regulating negative emotions. During routine health education, medical staff typically hold a dominant position, and patients may have limited opportunities to make decisions for themselves. However, through empowerment education (EE), patients can gain the tools and knowledge needed to take an active role in maintaining and improving their health. This includes the ability to monitor and manage their disease, minimize the impact of the disease on their social functioning, emotions, and relationships, and persistently treat their disease. The results of our study demonstrated a consistent trend between the changes in self-management and self-efficacy among liver transplant patients. This finding is supported by previous studies, which have shown that enhancing patients’ self-efficacy can improve their motivation for self-care and their ability to manage their health [38]. According to Bandura’s theory of self-efficacy, there is a dynamic relationship between an individual’s self-efficacy and their level of behavior, which interact and mutually reinforce each other [9]. Therefore, developing effective interventions to enhance patients’ self-efficacy can lead to improvements in their self-management abilities.

Attrition bias is a well-known concern in randomized controlled trials, as participants may drop out or withdraw from the study for a variety of reasons. In our study, we performed analysis per protocol, which meant that only those participants who completed the intervention were included in the final analysis. However, this approach may have increased the risk of attrition bias, leading to an overestimation of the intervention’s effect. To minimize this bias, we provided incentives and used reminder systems to encourage participants to complete the study. In future studies, we suggest using intention-to-treat analysis or conducting sensitivity analyses to minimize the risk of bias.

### Study limitation

There are some potential limitations to this study that need to be acknowledged. Firstly, this was a single-center trial, and due to factors such as staffing, material resources, and time constraints, the sample size was limited. Therefore, the generalizability of the results may be limited, and further studies with larger sample sizes and multiple centers are needed to confirm the findings. Secondly, the COVID-19 pandemic may have affected the study, leading to changes in patient care and follow-up, which may have influenced the results. Thirdly, although efforts were made to minimize contamination between the intervention and control groups, communication between patients in different groups was unavoidable, potentially affecting the outcomes. Finally, it should be noted that the analysis per protocol approach used in our study may increase the risk of attrition bias. To address this issue in future research, it may be helpful to recruit more participants than necessary for the sample size and minimize the number of follow-ups required.

## Conclusion

The results of this study suggest that empowerment education is an effective means of improving the self-management and self-efficacy of liver transplant patients, with better outcomes compared to routine health education. These findings have important implications for nursing practice and provide valuable guidance for clinical education of liver transplant patients.

## Data Availability

The datasets used and analyzed during the current study are available from the corresponding author on reasonable request.

## Abbreviations

CLTR: China Liver Transplant Registry
EEG: Empowerment education group
CG: Control group
χ2: Chi-square test
M ± SD: Mean ± standard deviation
CNY: Chinese Yuan
